# Notes and News

**Published:** 1903-06-13

**Authors:** 


### Notes and News.

The Princess Christian has opened the new Cottage Hospital at Caterham, erected as a local memorial to Queen Victoria. It provides for twelve patients in two wards. The architect is Mr. Ballantine, and the cost of the building has been £4,000.

The programme for the visit of the King and Queen to the new out-patients' department of the London Hospital included an inspection of the department. The Queen has herself given £1,000 to the present appeal, and continues to take a great interest in the Finsen Light department, which she initiated, and for which she has done so much.

The Metropolitan death-rate has been exceptionally low during the last few weeks. The total Metropolitan death-rate for the past year has been below the average.

The arrangements to convert a portion of Osborne House into a convalescent home for naval and military officers is progressing, and Captain Powell, R.N., has been appointed governor of the whole of the establishment.

Through the death of a woman in Kentish Town, attention has been called to the insanitary state of the mews in which she lived. It would be well for the health of London generally if this incident led to a reform in the matter of street and courtyard cleaning between Saturday night and Monday or Tuesday.

Sir William Ramsay received the gold medal awarded by the German Chemical Society at Berlin, for distinguished chemical research work. This medal is awarded once in five years to a foreigner of sufficient distinction to merit the award. It was instituted in 1888, and this is the first presentation which has been made. The medal was also given to Professor Moisson, of Paris. Sir William Ramsay's medal bears the inscription "For Distinguished Work in the field of General Chemistry and particularly for the Discovery of new Ingredients in the Air."

In Dublin six cases of concealed small-pox have been discovered; one patient was already recovering, so that the extent of the mischief cannot yet be estimated. In London the number of cases under treatment shows a slight increase.

An amusing defence was put in by a grocer whose wife was charged with selling adulterated cocoa. He said that she had neuralgia in the head, and that people suffering from this did not know what they were doing. But the magistrate failed to see that the presence of cane sugar and foreign starch in the cocoa was the natural result of neuralgia and administered a fine.

The Medical, Surgical, and Hygienic Exhibition at Queen's Hall proved most successful and popular. It was largely visited by members of the medical profession, who found a good deal to interest them conveniently exhibited under one roof. There is no doubt that these exhibitions serve a useful purpose in bringing to the notice of busy medical men much that is useful, but that otherwise would not easily obtain their attention.

At Davos, where it is proposed to erect the Queen Alexandra Sanatorium for Consumptives, the German Sanatorium established on the same lines has met with the greatest success. The applications for admission have so largely exceeded the accommodation available that there is little doubt that, in spite of the fact that the German institution is about to be enlarged, the Queen's Sanatorium will find plenty of useful work to be done.

An action was recently brought against the Apollinaris Water Company because salt and carbonic acid gas are introduced into the water described as "Natural" before bottling. But as it was shown that the water so treated was of the same constituency and character as that in the spring at a depth of 50 or 60 feet, it was held that the description of the water was practically a true one, and that no deception was practised on the public. This decision is important, as it defines for practical purposes the conditions which must be complied with if any alterations are made to a natural product.

The Report of the Health of the City of Birmingham, published by the Health Committee, and compiled by Dr. Hill, shows that the death-rate has fallen to 18 per 1,000, this being the lowest on record in that city. This fall exists in spite of an increase in the cases of scarlet fever from 3,389 to 5,044, and diphtheria from 533 to 787, respectively. This proportionate increase suggests that some rather intimate relation exists between the two diseases, of which in each case the mortality averages 17 per cent. In considering the epidemic diarrhœa in children and the prophylaxis of consumption, Dr. Hill takes every opportunity to condemn the small, badly-ventilated, back-to-back houses in the most insanitary quarters of Birmingham, and he recommends as one of the most important questions in the crusade against consumption, that modern dwellings be constructed for housing the poorer classes, and that every attention be paid to the air-space and ventilation.

